# Research on Seasonal Disease Warning Methods for Northern Winter Sheep Based on Ear-Base Temperature

**DOI:** 10.3390/ani16020344

**Published:** 2026-01-22

**Authors:** Jianzhao Zhou, Runjie Jiang, Dongsheng Xie, Tesuya Shimamura

**Affiliations:** 1 Faculty of Information Science and Engineering, Saitama University, Saitama 338-8570, Japan; zhou.j.590@ms.saitama-u.ac.jp; 2 College of Electrical and Information Engineering, Northeast Agricultural University, Harbin 150030, China; runjiejiang20@gmail.com (R.J.); 19854250790@163.com (D.X.)

**Keywords:** disease warning, ear base temperature, automatic monitoring, winter sheep breeding, low cost

## Abstract

The temperature at the base of the ear is highly correlated with the core body temperature of sheep and responds sensitively to febrile conditions, making it a valuable indicator of sheep health. In northern China, the closed housing environment during winter increases the incidence of seasonal diseases such as upper respiratory infections and pneumonia, which severely affect the economic efficiency of sheep farming. To address this issue, this study proposes an early-warning method for winter diseases in sheep based on ear-base temperature. Ear temperature, body weight, and environmental data were collected, and Random Forest was employed for feature selection. Bayesian optimization was used to fine-tune the hyperparameters of a one-dimensional convolutional neural network to construct a predictive model of ear-base temperature using data from healthy sheep. Based on the predicted normal range, an early-warning strategy was established to detect abnormal temperature patterns associated with disease onset. Experimental results demonstrated that the proposed method achieved a high detection rate for common winter diseases while maintaining a low false positive rate, and validation experiments confirmed its effectiveness under practical farming conditions. Combined with low-cost temperature-sensing ear tags, the proposed approach enables real-time health monitoring and provides timely early warnings for winter diseases in large-scale sheep farming, thereby improving management efficiency and economic performance.

## Simple Summary

Monitoring the health of sheep during winter is challenging, especially in northern regions where animals are kept in closed housing and respiratory diseases are common. Changes in body temperature often occur before visible clinical symptoms appear. In this study, we found that the temperature at the base of the ear could be used as an early indicator of disease in sheep. By continuously monitoring ear temperature together with body weight and environmental conditions, we established a method to identify abnormal temperature changes that may signal the early onset of disease. The proposed approach was tested under practical farming conditions and showed a high ability to detect winter diseases while avoiding false alarms. Combined with low-cost smart ear tags, this method provides a practical tool for real-time health monitoring and early disease warning in large-scale sheep farming.

## 1. Introduction

In northern China, winter sheep production typically relies on enclosed barns where poor ventilation facilitates disease outbreaks and transmission [1]. Extended recovery times in such environments increase veterinary costs. Early disease detection therefore improves cure rates, limits spread, and minimizes productivity loss, while also reducing antibiotic use and supporting eco-friendly livestock production.

Core body temperature is a primary indicator of fever [2] and is commonly measured with mercury or digital rectal thermometers. However, rectal measurement requires skilled handling of each animal and carries a risk of iatrogenic transmission [3]. Moreover, the physiological range of core temperature in sheep is narrow (38.5–40.5 °C) [4], which makes prediction sensitive to small errors [5]. By contrast, surface temperature responds more rapidly to thermal environmental changes [6,7], providing an immediate snapshot of physiological status. Studies in humans and animals show that surface temperature is informative for thermal sensation and disease symptomatology [8,9,10]. As a prominent thermal window, the ear-base temperature (EBT) is a practical indicator for health assessment in sheep. This is because the ear-base region is characterized by thin skin, abundant blood vessels, and relatively sparse hair coverage, enabling it to rapidly reflect changes in core body temperature, particularly under febrile conditions [11].

Models have been proposed to estimate rectal temperature from surface temperatures measured at body sites [12,13,14], and heat-window regions such as the ear base, eye, and udder often correlate best with rectal temperature [2]. Sasaki reported that surface temperature can vary with piglet age [15]. When using EBT to evaluate health, the interplay among age (or growth stage), activity [16], stress response [17], environment, and individual status must be considered [18]. In sheep, body weight is commonly used to represent growth stage; accordingly, we treat weight as a key covariate for EBT [19]. In addition, healthy pigs exhibit diurnal variation in surface temperature [20]; given the exposure of sheep to cold air, we emphasize outdoor daytime activity data in this work.

Automation of EBT monitoring has been enabled by smart ear tags and infrared thermography [21]. These non-contact or minimally invasive approaches preserve normal behavior and reduce stress responses [22]. Lu used overhead thermal images to extract piglet EBT and accurately identify febrile animals [18]. For sheep, however, wool can obscure infrared views of the ear base and frequent crowding complicates image acquisition [23,24], motivating the use of temperature-sensing ear tags in this study.

Recent livestock research increasingly integrates physiological, behavioral, and environmental information. For example, posture and movement features have been used to classify broiler health [25], chicken vocalizations have supported real-time surveillance of avian influenza [21], and image-based posture detection has aided early disease detection in pigs [26]. In addition, recent studies have examined the use of multi-dimensional data tools for livestock health and welfare management, highlighting the integration of health-related data and early-warning systems on commercial farms. For example, stakeholder perspectives in pig farming emphasize the value of data recording and early-warning tools that combine behavioral and health indicators for managing respiratory and gastrointestinal diseases [27]. Environmental monitoring technologies have also advanced, with wireless sensor networks and detection methods being developed to continuously assess environmental factors in livestock and poultry houses, such as temperature, humidity, and gas concentrations, which are important for animal welfare and health [28]. Furthermore, precision livestock research continues to explore comprehensive data-driven approaches for monitoring physiological indicators, such as activity levels, body temperature, and environmental conditions, to support health assessment and decision support in smart farming systems.

Long-term environmental monitoring has revealed relationships between air-quality parameters and animal health [29], and multi-factor environmental comfort evaluation has been implemented via an analytic hierarchy process [30]. Wearable multi-sensor systems for sheep can combine environmental and physiological information, including body temperature [31], heart rate [32], and respiratory rate [33], to predict comfort and reduce disease risk, although device damage remains a practical challenge in dense housing [34,35]. Similarly, production records coupled with meteorological data have been used to infer dairy health status [36], and environmental variables have been linked to pig body temperature [37]. Collectively, these findings support combining environmental and physiological signals for robust health assessment.

Building on this literature, this study aims to develop a robust early-warning framework for winter diseases in sheep by establishing ear-base temperature (EBT) as a health indicator that jointly models physiological (body weight) and environmental covariates. Random Forest is used for feature selection, and a 1D CNN, whose hyperparameters are tuned via Bayesian optimization, predicts healthy EBT. The predicted healthy range (95% confidence interval) is then compared with observed EBT to assess health status and trigger early warnings.

## 2. Materials and Methods

### 2.1. Experimental Design

All procedures were approved by the Animal Care and Use Committee of Northeast Agricultural University (Harbin, Heilongjiang, China) and conformed to institutional guidelines. Guided by prior studies and winter housing practices for commercial sheep farms in northern China, we collected environmental data [38] and physiological data from Hu sheep [39,40,41].

#### 2.1.1. Experimental Subjects and Site

**Figure 1:**
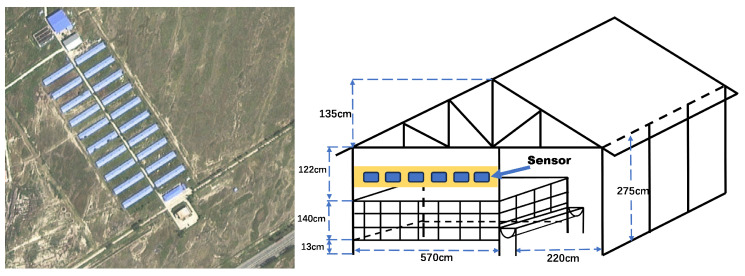
Satellite image of the pasture and regional structure of the sheep house.

Data were collected from 80 fattening Hu sheep (15–55 kg) at the WoYuan Livestock Breeding Farm in Zhaozhou County, Daqing City, Heilongjiang Province, China ($45.39^{\circ }\;N,\;125.04^{\circ }\;E$). The farm comprised 20 housing units (lamb pens, pregnant-ewe pens, sick pens, and standard pens), a burial area for mortalities, a veterinary medical-waste facility, and feed storage areas. The sheep were fed pelleted total mixed rations (TMR), and the ration composition is summarized in Table 1. Each housing area (total: 3624.31 $m^{2}$) included an outdoor activity space, an indoor resting area, feed troughs, and cart paths. The indoor resting area used a bamboo-slatted floor elevated 13 cm above the ground to facilitate manure drainage and cleaning. A satellite view of the farm and a cross-section of the housing structure are shown in Figure 1. Environmental sensors were installed at a distance of about 1.2 m from the sheep to prevent damage caused by trampling, jumping, or biting.

#### 2.1.2. Data Acquisition

**Figure 2:**
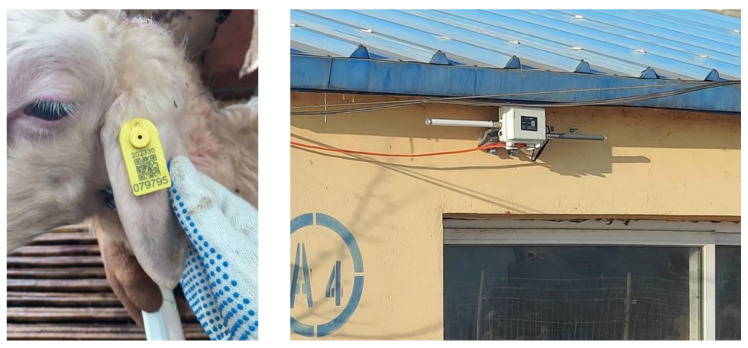
Smart ear tags (http://www.fofia.com/products/proxq/208.html, accessed on 11 February 2025) and gateways.

**Figure 3:**
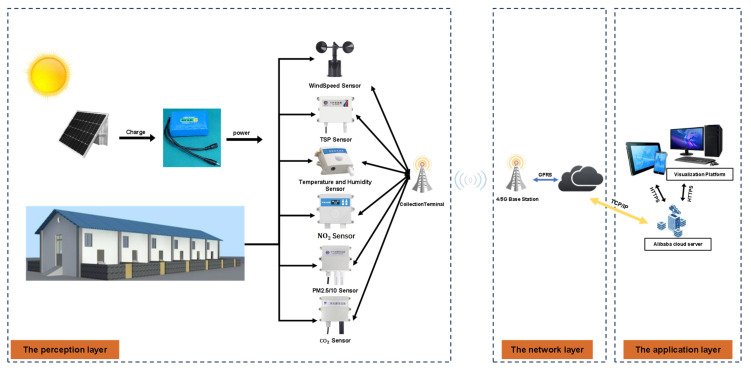
Environmental monitoring system.

The study spanned the peak winter-disease season (15 November 2024–21 February 2025). EBTs were monitored using ET960 smart ear tags (Wuxi Fuhua Company, Wuxi, Jiangsu, China), which served as the core sensing device in this study. The tags are designed for continuous monitoring of ear-base temperature in sheep and are fixed to the ear in a manner similar to conventional livestock ear tags, requiring penetration of the ear tissue for stable attachment. A built-in temperature sensor is positioned at the ear base, enabling continuous acquisition of peripheral temperature signals associated with thermoregulation and health status. The tags were affixed as shown in Figure 2; data were transmitted to a server via a gateway. Environmental parameters (air temperature, relative humidity, ammonia, etc.) were acquired by an IoT-based monitoring system with 10 min sampling, solar power, and a 4G uplink for cloud visualization on PC and mobile devices (see the system architecture in Figure 3). Sensor response time was ≤30 s; repeatability was ±2%; linear error was ±2%; and zero drift was ±1%. Key specifications are summarized in Table 2. Outdoor solar radiation (MJ·m^−2^) was obtained from the China Meteorological Data Network. Manure in the sheep barn was removed every two days. During the cleaning process, the sheep were driven into another vacant pen. A weighing scale was installed beneath a narrow passageway, enabling individual body weight measurements to be recorded for each sheep as they passed through during herding.

In total, 82,412 healthy-sheep records were collected to train the EBT prediction model, and 14,797 sick-sheep records were used to validate the early-warning method (Table 3). Health-status labels were determined based on clinical diagnoses by on-site veterinarians and routine hematological examinations were conducted every 10 days [38].

#### 2.1.3. Data Processing

**Figure 4:**
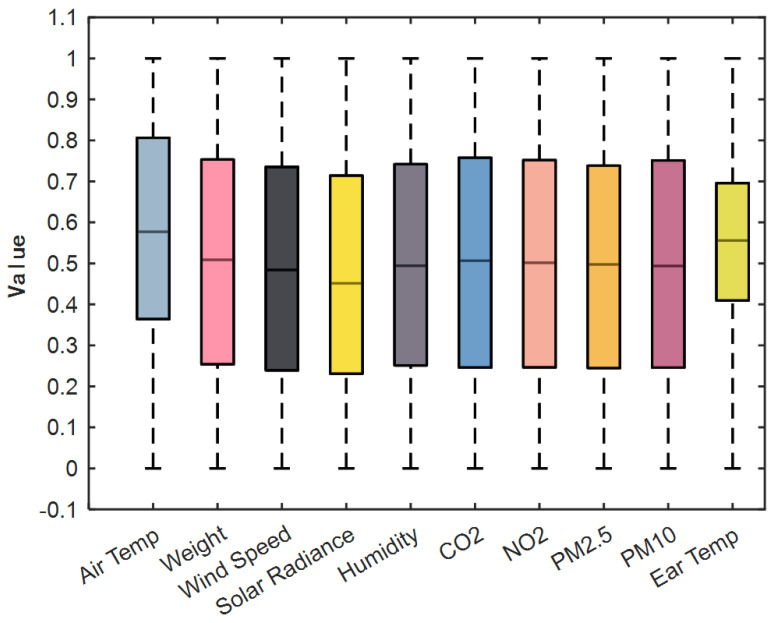
Quartile method to discriminate outlier box plot.

Environmental data transmission may be affected by packet loss, transmission delays, and occasional duplicated or corrupted packets during GPRS communication. We therefore applied the interquartile range (IQR) method to detect and remove anomalies. For each variable, after sorting the observations, we computed the first ($Q_{1}$) and third ($Q_{3}$) quartiles and defined the normal range as $\left[Q_{1}-1.5\times IQR,\;Q_{3}+1.5\times IQR\right]$, where $IQR=Q_{3}-Q_{1}$. Values outside that interval were labeled as outliers and removed. To improve convergence and accuracy during training, variables with differing units were normalized. After preprocessing, a total of 82,977 samples were obtained and chronologically divided into training, validation, and test sets, with a typical ratio of 70% for training, 15% for validation, and 15% for testing; the distribution is illustrated in Figure 4.

(1) $$Q_{1}=x_{{\left(0.25\times N\right)}^{*}}$$

(2) $$Q_{3}=x_{{\left(0.75\times N\right)}^{*}}$$

(3) $$IQR=Q_{3}-Q_{1}$$

### 2.2. Predictive Model Construction

#### 2.2.1. Random-Forest Feature Selection

There is a complex interaction among ear temperature in fattening sheep, environmental factors, and body weight. Because a wide range of parameters is involved, redundancy and information overlap may occur. The ensemble learning algorithm based on decision trees, Random Forest (RF), is well suited to address this issue through the selection of important features [42]. Therefore, this study employed RF to select and evaluate the key features affecting sheep ear temperature, with the goal of optimizing the model architecture and enhancing predictive performance.

RF calculates feature importance primarily by evaluating each feature’s contribution to the decision trees within the ensemble and comparing these contributions to obtain a ranking of feature importance. Typically, the out-of-bag (OOB) error rate is used as the evaluation metric for feature selection, as shown in Equation (4). This approach makes it possible to identify the most influential features by assessing their impact on prediction accuracy.

(4) $${\mathrm{FIM}}_{i}=\frac{\sum (\mathrm{errOO}B_{2}-\mathrm{errOO}B_{1})}{N}$$

#### 2.2.2. One-Dimensional CNN Model

A one-dimensional convolutional neural network (1D CNN) is a variant of CNN in which the convolutional kernels operate along a single dimension [43]. It has strong feature-extraction capability for regression tasks and is well suited for modeling ear-base temperature as a continuous physiological variable: alternating convolutional and pooling layers capture nonlinear features from the raw data, while fully connected layers facilitate adaptive feature learning. The 1D CNN approach addresses the time-consuming manual feature extraction required in traditional methods, enabling end-to-end information processing.

**Figure 5:**
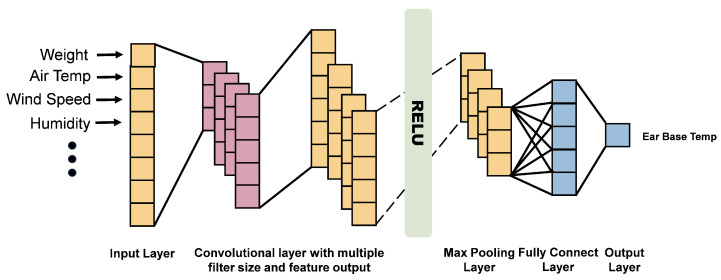
One-dimensional CNN network structure diagram.

The basic structure of the 1D CNN is illustrated in Figure 5. It comprises an input layer, several convolutional layers, several pooling layers, a fully connected layer, and an output layer. Environmental parameters and body weight are passed to the first convolutional layer in the form of a one-dimensional vector. Each convolutional kernel in the convolutional layer slides along the input vector to extract features related to ear-base temperature (EBT). After being processed by an activation function, these features undergo pooling operations before being passed to the next convolutional layer. The output of the *f*th convolutional kernel in the *i*th convolutional layer can be expressed as Equation (5).

(5) $$y_{f}^{\left(i\right)}=\mathrm{Conv}\left(x^{\left(i-1\right)},w_{f}^{\left(i\right)}\right)+b_{f}^{\left(i\right)}$$

In Equation (5), $\mathrm{Conv}\left(x^{\left(i-1\right)},w_{f}^{\left(i\right)}\right)$ represents the convolution operation between the input $x^{\left(i-1\right)}$ and the convolution kernel $w_{f}^{\left(i\right)}$, where $b_{f}^{\left(i\right)}$ is the bias of the *f*th convolutional kernel. The size *r* of the one-dimensional feature vector output by each convolutional kernel is determined by the kernel size *m*, the number of elements *q* in each sample, and the convolution stride *n*, as shown in Equation (6).

(6) $$r=\frac{q-m}{n}+1$$

The powerful feature-extraction capability of CNNs stems from their multi-layer architecture, which includes convolutional and pooling layers. However, as the network deepens, the number of learnable parameters increases, making the model more complex. In addition, hyperparameters such as the number of convolutional layers, kernel sizes, and the number of filters must be set manually. These hyperparameters have a substantial impact on the model’s performance, convergence speed, and stability.

To improve the predictive accuracy, generalization ability, and robustness of the 1D CNN, we employed Bayesian optimization to fine-tune these hyperparameters. Bayesian optimization efficiently searches for near-optimal settings by balancing exploration and exploitation, thereby accelerating the hyperparameter-tuning process and enhancing overall model performance.

#### 2.2.3. Bayesian Hyperparameter Optimization

The effectiveness of CNN training is influenced by several hyperparameters. Manually adjusting these hyperparameters can be time-consuming, and the results are often subject to personal bias, making it difficult to find an optimal combination. As convolutional neural networks continue to develop and become more complex across various applications, tuning these parameters becomes even more challenging. Traditional grid-search methods, which require experimenting with all possible hyperparameter combinations, are computationally expensive and time-consuming.

Bayesian optimization, as summarized in Table 4, offers a more efficient solution based on black-box optimization. Its core idea is to search for the minimum value of the model’s loss function. By iteratively updating its belief about the objective function, Bayesian optimization selects the next set of hyperparameters more strategically, thereby significantly reducing the number of experiments required to find a near-optimal combination.

In this context, *X* represents the hyperparameter search space, and *f* is the target model to be optimized. The dataset *D* consists of multiple pairs $\left(x_{i},y_{i}\right)$, where $x_{i}$ represents a hyperparameter configuration, and $y_{i}$ is the corresponding loss value of the CNN. The term *T* denotes the number of iterations, and *S* stands for the acquisition function (AF). The model *M* is a probabilistic surrogate fitted to the dataset *D*; in this study, we used Gaussian process regression (GPR) for this purpose.

**Figure 6:**
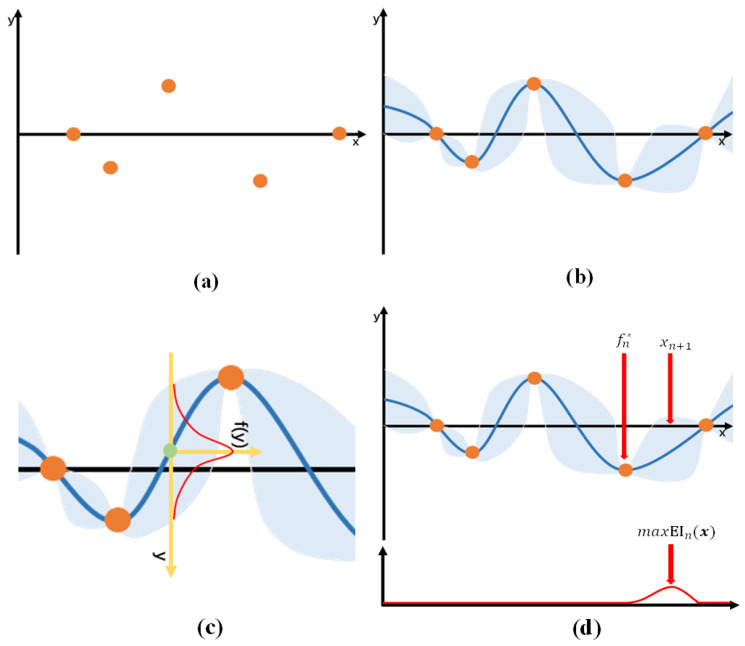
Schematic diagram of the Bayesian optimization process. (**a**) Random sampling of hyperparameter combinations, (**b**) Modeling the hyperparameter distribution using a Gaussian process, (**c**) Gaussian distribution corresponding to the given hyperparameters, (**d**) Acquisition of the next sampling point.

Initially, several random hyperparameter combinations $x_{i}$ are sampled from the search space *X* and evaluated by the objective function, yielding loss values $y_{i}=f\left(x_{i}\right)$. These initial $\left(x_{i},y_{i}\right)$ samples are then added to the dataset *D* (as illustrated in Figure 6a), and the optimization is configured to run for *T* iterations. A Gaussian process is used to model the distribution of the objective function (Figure 6b). For a given hyperparameter combination *x*, the CNN loss value is assumed to follow a Gaussian distribution with mean μ and variance $\sigma^{2}$ (Figure 6c, Equation (7)). The function f(y) denotes the probability density function (PDF) of the loss value *y* (Equation (8)).

(7) $$p\left(y|x,D\right)=N\left(y|\mu ,\sigma^{2}\right)$$

(8) $$f\left(y\right)=\frac{1}{\sqrt{2\pi }\sigma }\exp\;\left(-\frac{{\left(y-\mu \right)}^{2}}{2\sigma^{2}}\right)$$

Next, we use an acquisition function to select new observation points in order to improve upon the fitted stochastic function. The function u(x) represents the improvement associated with evaluating *x* as a candidate next observation point (Equation (9)). Let $f_{n}^{*}$ denote the minimum loss value observed among the current sample points (Equation (10)). If f(x) at *x* is less than $f_{n}^{*}$, the difference $f_{n}^{*}-f\left(x\right)$ is taken as the improvement. We then select the point *x* that yields the maximum improvement value as the next observation point (Figure 6d).

(9) $$u\left(x\right)=\max\;\left(0,\;f_{n}^{*}-f\left(x\right)\right)$$

(10) $$f_{n}^{*}=\min\;\left(f\left(x_{1}\right),\ldots ,f\left(x_{n}\right)\right)$$

Based on the initial set of *n* observation points, we obtain the probability distribution of f(x) using a Gaussian process model. This allows us to compute the expected improvement $EI_{n}\left(x\right)$ for all *x* (Equation (11)). Here, E[·] denotes the conditional expectation given the existing *n* sampled points $x_{1:n}$ and their corresponding loss values $y_{1:n}$ (Equation (12)). The probability distribution required to evaluate this expectation is provided by the Gaussian process regression described above.

(11) $${\mathrm{EI}}_{n}\left(x\right)=E_{n}\;\left[{\left(f\left(x\right)-f_{n}^{*}\right)}^{+}\right]$$

(12) $$E_{n}\left[\cdot \right]=E\;\left[\;\cdot \;|\;x_{1:n},\;y_{1:n}\right]$$

Finally, the hyperparameter combination that maximizes the improvement value is selected as the next observation point $x_{n+1}$ (Equation (13), Figure 6d)

(13) $$x_{n+1}=\arg\max{\mathrm{EI}}_{n}\left(x\right)$$

The observation point $x_{i}$ obtained from the acquisition function is used to train the model. After training, the corresponding loss value $y_{i}$ is computed, and the pair $\left(x_{i},y_{i}\right)$ is added to the dataset *D*. This new sample then serves as additional data for the Gaussian process regression in the next iteration. Once the number of iterations reaches *T*, the hyperparameter combination associated with the minimum loss value in the dataset *D* is selected, completing the optimization process.

#### 2.2.4. Evaluation Metrics

In regression modeling, evaluation metrics based on the predicted and actual values are typically used to assess model performance. In this study, we employed mean absolute error (MAE, Equation (14)), mean absolute percentage error (MAPE, Equation (15)), root-mean-square error (RMSE, Equation (16)), and the coefficient of determination ($R^{2}$, Equation (17)).

(14) $$\mathrm{MAE}=\frac{1}{n}\sum_{i=1}^{n}\left|y_{i}-{\hat{y}}_{i}\right|$$

(15) $$\mathrm{MAPE}=\frac{1}{n}\sum_{i=1}^{n}\frac{|y_{i}-{\hat{y}}_{i}|}{|y_{i}|}$$

(16) $$\mathrm{RMSE}=\sqrt{\frac{1}{n}\sum_{i=1}^{n}{\left(y_{i}-{\hat{y}}_{i}\right)}^{2}}$$

(17) $$R^{2}=1-\frac{\sum_{i=0}^{n}{\left(y_{i}-{\hat{y}}_{i}\right)}^{2}}{\sum_{i=0}^{n}{\left(y_{i}-\bar{y}\right)}^{2}}$$

In these equations, *n* represents the number of samples, $y_{i}$ denotes the actual sample values, $\bar{y}$ is the mean of the actual sample values, and ${\hat{y}}_{i}$ stands for the predicted values. These quantities were used to calculate the evaluation metrics, which assessed the model’s predictive performance relative to the observed data.

#### 2.2.5. Experimental Environment

The model developed in this study was trained on a computer equipped with an Intel i5-9400 2.90 GHz CPU and an NVIDIA GeForce RTX 1660 Ti GPU. The operating system was Windows 11, and all models were implemented in MATLAB R2022a.

#### 2.2.6. Disease Early-Warning Method Based on Ear Temperature

**Figure 7:**
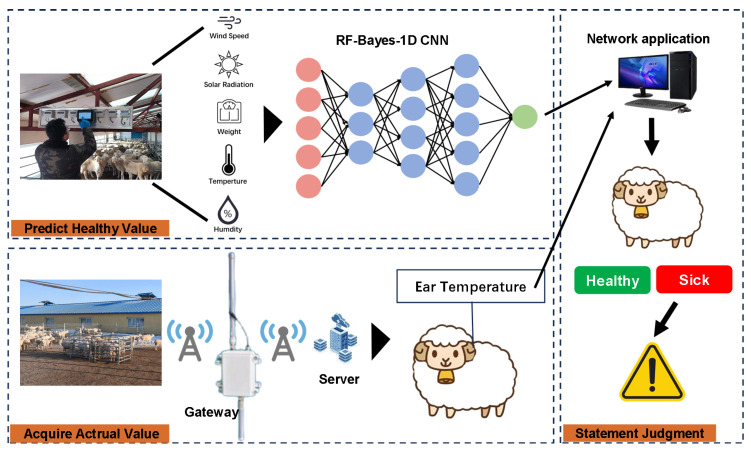
Structure of the disease early-warning method.

The structure of the disease early-warning method based on ear-base temperature (EBT) is illustrated in Figure 7. In the IoT-based environmental monitoring system, environmental parameters were collected by monitoring devices and, together with sheep body weight (we calculated the average daily gain based on bi-daily periodic weight measurements and the feed conversion ratio (FCR=4.35), with the estimation error of daily gain maintained within ±5–9%.), were fed into the model to estimate the normal EBT of the sheep. The ear-tag thermometer recorded the actual ear-base temperature and transmitted it to the server via a gateway. By comparing the predicted and measured EBT values, the health status of the sheep could be assessed, and an early warning was issued if any abnormalities were detected.

In this study, the monitored sheep’s body weight, ambient temperature, wind speed, solar radiation intensity, and relative humidity were combined into a one-dimensional input vector $x_{\mathrm{new}}$ for the RF–Bayes–1D CNN model. The model outputted the predicted healthy ear temperature ${\hat{T}}_{\mathrm{ear},\mathrm{new}}$. The 95% confidence interval $CI_{95\%}$ (Equation (18)) was then calculated as the acceptable range for healthy ear temperature, where $t_{0.025}$ is the critical value from the *t*-distribution with the appropriate degrees of freedom, and $SE_{\mathrm{pred}}$ is the standard error of the prediction. In Equation (19), *X* denotes the concatenated matrix of training samples, and $\sigma_{\mathrm{res}}^{2}$ is the variance of the model residuals.

(18) $$CI_{95\%}={\hat{T}}_{\mathrm{ear},\mathrm{new}}\pm t_{0.025}\times SE_{\mathrm{pred}}$$

(19) $$SE_{\mathrm{pred}}=\sqrt{\sigma_{\mathrm{res}}^{2}\left[1+x_{\mathrm{new}}^{T}{\left(X^{T}X\right)}^{-1}x_{\mathrm{new}}\right]}$$

Based on the confidence interval $CI_{95\%}$ and the RF–Bayes–1D CNN (RBC) model, a disease warning model was established (Equation (20)).

(20) $$f\left(\mathrm{Features},\mathrm{EarBaseTem}\right)=\left\{\begin{array}{cc} \mathrm{Healthy}, & \mathrm{if}\;\mathrm{EarBaseTem}\in CI_{95\%}\left(\mathrm{RBC}(\mathrm{Features})\right)\\ \mathrm{Warning}, & \mathrm{if}\;\mathrm{EarBaseTem}\notin CI_{95\%}\left(\mathrm{RBC}(\mathrm{Features})\right) \end{array}\right.$$

## 3. Results

### 3.1. Random Forest Feature Importance Selection

To identify the key factors influencing the thermal window region of sheep ears, we employed the RF algorithm to calculate the importance of nine input parameters. The ranking of these features is presented in Table 5. To verify the effectiveness of the RF-based feature ranking, we incrementally incorporated the features in order of importance and adjusted the input layer size and network structure accordingly. After training the corresponding models, the Mean Absolute Error (MAE) values were obtained, as shown in Table 6.

With a single input feature, the model had the smallest input dimension but exhibited poor fitting performance, resulting in the highest MAE. When three input features were used, the input dimension was still relatively small, and the information remained limited, so the model provided only moderate fitting performance, although the MAE was reduced compared with the single-feature case.

With five input features, the MAE decreased further, indicating improved fitting performance while maintaining a relatively compact input dimension. However, when six to eight input features were included, redundant information began to degrade the model’s predictive performance.

### 3.2. Bayesian Hyperparameter Optimization Settings and Results

After selecting the input features and determining the input-layer dimension and the number of output nodes for the 1D CNN model, we applied Bayesian optimization to tune the hyperparameters, including the kernel size, number of convolutional layers, number of kernels, initial learning rate, momentum factor (i.e., the influence of the previous iteration on the current update), and regularization coefficient. After 60 optimization iterations, we obtained the hyperparameter combination summarized in Table 7. This combination was then used to construct the final 1D CNN model.

### 3.3. Construction Results of RF–Bayes–1D CNN and Comparison of Methods

**Figure 8:**
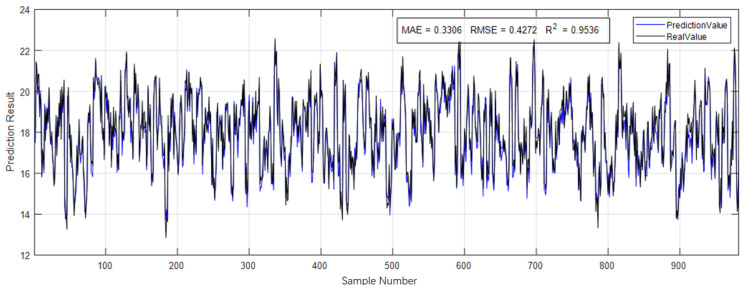
Model performance on the test set.

We constructed the 1D CNN architecture based on the hyperparameter combination obtained from Bayesian optimization and trained the model using the high-quality dataset processed in Section 2.1.3. The dataset was split into training, validation, and test sets with a ratio of 7:1.5:1.5. On the test set, the final performance of the RF–Bayes–1D CNN model was as follows: MAE = 0.3306, MAPE = 0.0176, RMSE = 0.4272, and $R^{2}=0.9536$. The model’s performance on a subset of the test set is illustrated in Figure 8.

To validate the effectiveness of the RF–Bayes–1D CNN model proposed in this study for predicting the ear temperature of meat sheep, we also trained a Backpropagation Neural Network (BP) model and a standard CNN model using the same experimental dataset. For all three models, we applied three common hyperparameter optimization methods—grid search, Bayesian optimization, and random search—to tune their hyperparameters. To prevent overfitting and assess model robustness, k-fold cross-validation was employed during model training. The healthy sheep dataset was randomly divided into k folds, with each fold used once as the validation set while the remaining folds were used for training. Model performance is reported as the average across all folds. The performance of each method on the validation set is summarized in Table 8.

These results clearly show that hyperparameter optimization plays a crucial role in enhancing model performance. For the RF–Bayes–BP model, the MAE was 0.3895, indicating relatively poor performance. This suggests that for the BP model with a relatively small and low-complexity hyperparameter search space, Bayesian optimization does not provide a clear advantage. In such cases, grid search and random search can achieve comparable or even better performance.

By contrast, the RF–Bayes–1D CNN model achieved an MAE of 0.3306. For the CNN model, which has a relatively complex hyperparameter search space, Bayesian optimization significantly outperformed grid search, which relies on exhaustive evaluation over a predefined grid. In addition, for these higher-dimensional and more complex CNN hyperparameter optimization problems, random search, which samples hyperparameters uniformly without exploiting information from previous evaluations, was less effective than Bayesian optimization.

These findings demonstrate that Bayesian optimization is particularly advantageous for complex models with high-dimensional hyperparameter spaces, such as CNNs, whereas simpler models with low-dimensional search spaces may not benefit as much.

### 3.4. Disease Early-Warning Method Based on Ear Temperature

The model was validated using the sick-sheep dataset and achieved a correct identification rate of 97.6% for diseased animals. To further refine the disease-warning method for sheep based on the RF–Bayes–1D CNN model, 37 experimental sheep were divided into four groups (Table 9) according to differences in their body-temperature responses to disease. $\mathrm{Group}\;T_{1}$ consisted of healthy sheep as determined by the resident veterinarian, whereas $\mathrm{Groups}\;T_{2}$, $T_{3}$, and $T_{4}$ comprised sick sheep diagnosed by the veterinarian.

$\mathrm{Group}T_{2}$: Sheep showing early symptoms of non-febrile diseases, including diarrhea, rumen overload, and rumen bloat.
$\mathrm{Group}T_{3}$: Sheep exhibiting intermittent fever as an early symptom, with diseases such as mange, infectious gastritis, and various parasitic infections.
$\mathrm{Group}T_{4}$: Sheep presenting with prolonged fever due to diseases such as pneumonia and upper respiratory infections, which are also common in winter [1].

The experiment involved continuous data collection of environmental conditions, body weight, and ear temperature over a 90 min period. Environmental and weight data were fed into the RF–Bayes–1D CNN model to obtain the predicted healthy ear temperature. The actual ear temperature of each sheep was then compared with the predicted value and its corresponding healthy ear-temperature range, which was defined as the 95% confidence interval derived from the RF–Bayes–1D CNN model predictions.

**Figure 9:**
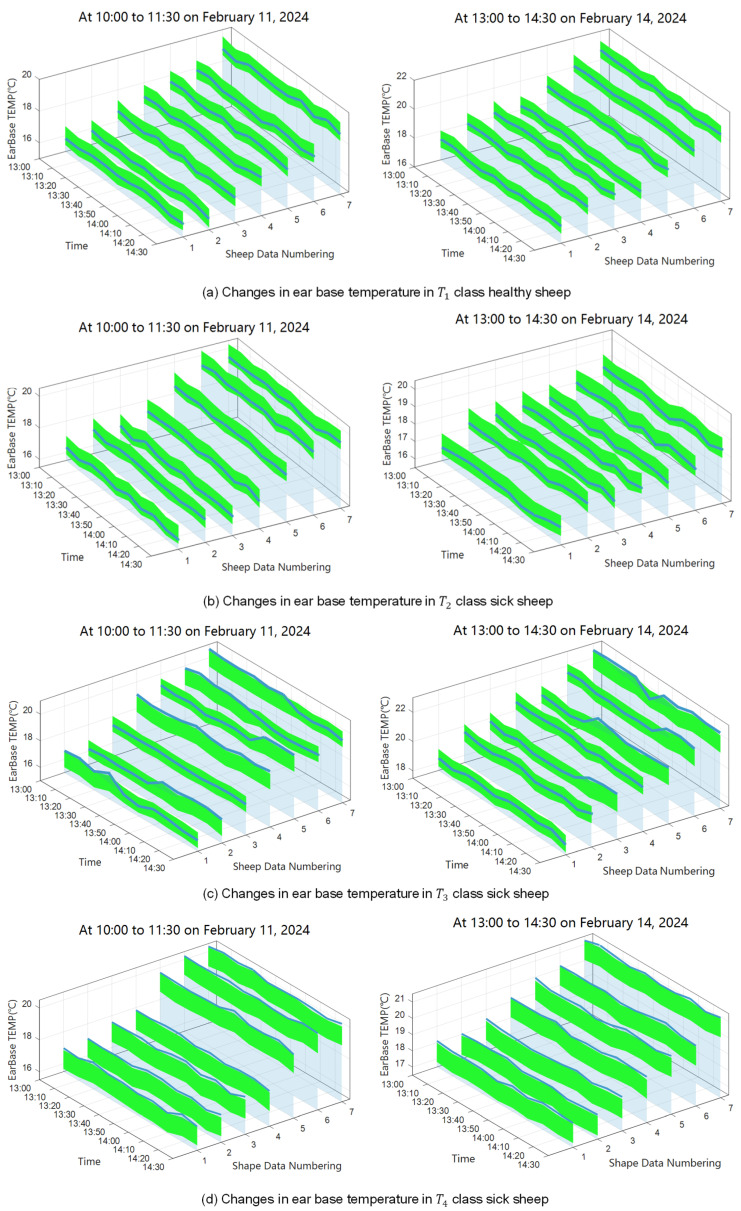
The identification effect of sick sheep data.

The experimental results are shown in Figure 9. For $\mathrm{Group}\;T_{1}$ (healthy sheep), ear temperature remained relatively stable within the defined healthy range (Figure 9a), meaning that the temperature fluctuations were moderate and did not approach the upper or lower thresholds of the healthy range, unlike the other three groups. The green band indicates the healthy range, and the blue solid line denotes the actual ear temperature. Instances in which the temperature exceeded the healthy range were rare.

$\mathrm{Group}\;T_{2}$ consisted of sheep exhibiting early-stage, non-febrile digestive disorders, including diarrhea, ruminal tympany (rumen flatus), and ruminal content accumulation. At this stage, animals showed no apparent fever, while mild gastrointestinal symptoms such as loose feces, reduced rumination, abdominal distension, and decreased feed intake were observed. Ear temperature occasionally dropped below the healthy range (Figure 9b), approaching the lower boundary of the normal interval.

In $\mathrm{Group}\;T_{3}$ (sheep with intermittent fever), ear temperature intermittently exceeded the healthy range (Figure 9c), with abnormal periods typically lasting 30–40 min, while shorter abnormal episodes of 10–20 min were relatively infrequent.

For $\mathrm{Group}\;T_{4}$ (sheep with prolonged fever), ear temperature frequently remained above the healthy range for extended periods (40–60 min) within the 90 min observation window (Figure 9d).

Therefore, we defined the warning criterion as an ear temperature remaining above the normal threshold for a continuous duration of 30 min, based on the observed duration of abnormal temperature episodes in $\mathrm{Group}\;T_{3}$ and *T*_4_, which were typically longer than transient fluctuations observed in healthy and non-febrile sheep. To further validate this conclusion, we randomly selected 35 sheep, tagged them for identification, and continuously monitored and analyzed their ear temperatures over a two-week period. The validation results showed that the method achieved an accuracy between 85.14% and 89.71% in diagnosing common diseases in sheep.

The main cause of misclassification was that sheep suffering from diseases such as diarrhea, gastroenteritis, parasitic infections, and lamb dysentery did not always exhibit marked fever, or their febrile episodes were infrequent and of short duration, making it difficult to assess these conditions accurately using ear temperature alone. However, for common winter diseases such as upper respiratory infections and pneumonia, which typically induce pronounced fever, the detection rate reached 95.7%, while the false positive rate in healthy animals was 5.3%.

## 4. Discussion

This study developed a disease early-warning method for winter-housed meat sheep in northern China based on ear-base temperature (EBT), integrating both environmental and physiological information into an RF–Bayes–1D CNN framework. The main findings were that (i) EBT under healthy conditions can be accurately predicted from a limited set of key environmental variables and body weight, (ii) the RF–Bayes–1D CNN model achieved substantially higher predictive performance than conventional neural network approaches, and (iii) a warning strategy built on deviations from the predicted healthy temperature range could effectively identify common winter diseases associated with fever in intensive sheep barns. Together, these results support the feasibility of using low-cost temperature-sensing ear tags, combined with IoT-based environmental monitoring, for continuous health surveillance of winter sheep.

The feature-selection results highlighted that air temperature, wind speed, solar radiation, relative humidity, and body weight were the most influential factors affecting EBT, whereas other variables such as PM_2.5_, PM_10_, CO_2_, and NH_3_ contributed little to the prediction. This is consistent with the thermoregulatory physiology of sheep: the thermal window at the ear base is strongly influenced by convective and radiative heat exchange with the environment, and by the animal’s size and metabolic heat production, which determine peripheral vasomotor responses and heat dissipation [44,45]. Recent studies also show that environmental conditions such as ambient temperature and airflow play a major role in modulating surface temperature and peripheral blood flow in livestock, affecting thermal comfort and heat exchange with the environment [46]. In poorly ventilated winter barns, even small changes in air temperature and airflow can markedly alter peripheral blood flow and surface temperature. The absence of strong multicollinearity among the selected variables suggests that each one provides complementary information, allowing the model to capture the combined effects of macro-climate, micro-environment, and individual characteristics on ear temperature.

From a modeling perspective, the integration of Random Forest-based feature selection with a Bayesian-optimized 1D CNN allowed the model to achieve high predictive accuracy (low MAE and RMSE and a high coefficient of determination, $R^{2}$) with a relatively compact input space. Compared with a BP neural network and a standard CNN tuned through manual or heuristic search, the RF–Bayes–1D CNN was better able to extract useful patterns from the time-series-like data and to generalize to unseen samples. The 1D CNN architecture is particularly suitable for this problem because it can automatically learn local temporal features from multi-channel input sequences, while Bayesian optimization efficiently explores hyperparameter combinations such as the number of layers, kernel sizes, number of filters, and learning rate. This approach not only improves predictive performance but also reduces the trial-and-error effort usually required to design deep-learning models for livestock applications.

On this basis, a disease early-warning method was established that compared the measured ear temperature with the predicted healthy range and triggered an alert when the value continuously exceeded the upper confidence limit for at least 30 min. The experimental grouping of sheep into healthy animals, individuals with early non-febrile symptoms, sheep with intermittent fever, and sheep with persistent fever clearly showed that the temporal behavior of ear temperature differed among health states. Specifically, the ear temperature of healthy sheep remained within the model-predicted 95% confidence interval for healthy ear temperature, defined by the lower and upper bounds ${\mathrm{CI}}_{\mathrm{lower}}$ and ${\mathrm{CI}}_{\mathrm{upper}}$, whereas sheep in diseased states frequently approached or exceeded the upper confidence limit. Healthy sheep remained largely within the normal range, whereas animals with sustained fever frequently exceeded the upper bound for extended periods within the 90 min observation window. Using this criterion, the system correctly identified the vast majority of diseased animals in the controlled trials and achieved an accuracy of approximately 85–90% in a two-week verification test on randomly selected sheep. For winter diseases that typically present with fever, such as respiratory infections and pneumonia, the recognition rate was particularly high. This is because these diseases induce a rapid and systemic febrile response associated with inflammatory cytokine release, which leads to increased core temperature and enhanced peripheral blood perfusion [46,47]. As a prominent thermal window, the ear base responds quickly to such changes in systemic thermoregulation, allowing fever-related temperature elevations to be captured more sensitively than in non-febrile or localized conditions, especially under intensive winter housing [48,49].

However, the study also revealed important limitations of relying solely on ear temperature for early disease warning. Diseases that do not produce a clear or sustained fever, such as some forms of diarrhea, ruminal disorders, parasitic infections, and lamb dysentery, were more likely to be misclassified. In such cases, abnormal ear-temperature responses may be short-lived or absent, and the requirement for a 30 min continuous exceedance can lead to under-detection. In addition, ear temperature can be influenced by transient factors such as acute stress, sudden environmental changes, or local vascular responses that are not directly related to systemic illness. Although the model partly compensates for these influences by incorporating environmental variables, false positives and false negatives cannot be completely avoided.

Several other limitations need to be considered when interpreting the results and applying the method in practice. First, all data were collected from Hu sheep on a single farm in one region and season. The generalizability of the model to other breeds, management systems, climatic conditions, and seasons remains to be validated. Second, although the dataset contained a large number of observations, the diversity of disease types and severity levels was limited, which may reduce the robustness of the warning system for rare or atypical conditions. Third, the proposed method depends on the stable operation of ear-tag sensors and the IoT infrastructure. Sensor failure, signal loss, or calibration drift could degrade performance if not monitored and corrected in real time.

Despite these limitations, the proposed RF–Bayes–1D CNN-based ear-temperature warning system has clear practical implications for precision sheep farming in cold regions. It offers a low-cost, non-invasive, and automated tool for continuous health monitoring, which can support earlier detection of disease, timelier veterinary intervention, reductions in mortality and economic losses, and more prudent use of antimicrobial drugs. In the longer term, integrating ear-temperature data with additional behavioral, production, and physiological indicators (such as activity, feeding and drinking behavior, rumination, and weight gain) could lead to more comprehensive multimodal health-monitoring platforms. Future work should also focus on multi-farm and multi-season validation, adaptive individual-based thresholds, and the integration of the warning system into farm decision-support software to facilitate rapid and targeted responses by farmers and veterinarians.

## 5. Conclusions

The present study proposed a disease early-warning method for winter meat sheep in northern China based on ear-base temperature, using a combination of environmental and physiological information within an RF–Bayes–1D CNN modeling framework. Physiological data from sheep and environmental data from an IoT-based monitoring system were collected under commercial winter-barn conditions and used to train and validate an ear-temperature prediction model. RF feature selection identified air temperature, wind speed, solar radiation, relative humidity, and body weight as key predictors of healthy ear temperature, enabling the construction of a compact yet informative input feature set.

Using these selected features, a 1D CNN model optimized by Bayesian methods was developed to predict healthy ear-base temperature with high accuracy. The RF–Bayes–1D CNN model outperformed traditional neural-network approaches in terms of MAE, RMSE, and $R^{2}$, demonstrating strong predictive performance and good generalization. On this basis, a disease early-warning strategy was established by defining an individual healthy temperature range and generating alerts when measured ear temperature continuously exceeded the upper threshold. The method achieved high recognition rates for common winter diseases that present with fever and showed stable performance in verification tests under practical farming conditions.

Overall, the proposed ear-temperature-based warning system provides a low-cost, automated, and non-invasive tool for early detection of disease in winter sheep farming, with potential benefits for animal welfare, economic efficiency, and rational use of antimicrobial agents. Future work should expand the dataset across different farms, regions, breeds, and seasons, and explore the integration of additional behavioral and physiological indicators to improve the detection of non-febrile diseases and to further enhance the robustness and applicability of the system.

## Figures and Tables

**Table 1:** Compositions of total mixed rations and conventional nutrients.

Project	Percentage
Dry Matter/% DM	87.39
Crude Protein/% DM	13.57
Crude Fat/% DM	5.93
Coarse Ash/% DM	10.70
Neutral Detergent Fiber/% DM	14.91
Neutral Detergent Fiber/% DM	31.42
Ca/% DM	1.09
P/% DM	0.51

**Table 2:** Testing Index and Measurement Details.

Testing Index	Measurement Range	Accuracy
Air temperature (°C)	−40~+60 °C	±0.2 °C
Air relative humidity (%)	0~80%RH	±2%RH
PM_2.5_ mass concentration (μg/m^3^)	0~999 μg/m^3^	±10%
PM_10_ mass concentration (μg/m^3^)	0~999 μg/m^3^	±10%
Wind speed (m/s)	0~32.4 m/s	±1 m/s
Solar radiance (W/m^2^)	0~2000 W/m^2^	±1 W/m^2^
CO_2_ (ppm)	0~10,000 ppm	±50 ppm
NO_2_ (ppm)	0~1000 ppm	±8%

**Table 3:** Information about environmental and physiological data from Hu sheep.

Parameter	Maximum	Minimum	Measure
Air temperature	−8.1	−26.8	°C
Air relative humidity	80.9	50	%
PM_2.5_	145	1	μg/m^3^
PM_10_	451	1	μg/m^3^
Wind speed	9.59	0.81	m/s
NO_2_	0.125	0.001	ppm
CO_2_	2672	488	ppm
Ear base temperature	22.4	12.5	°C
Solar radiance	306.1	41.2	J/m^2^
Weight	51.5	15	kg

**Table 4:** Bayesian optimization pseudocode.

1.	Input: f,X,S,M
2.	D←INITSAMPLES(f,X)
3.	For i←1 to *T* do
4.	p(y∣x,D)←FITMODEL(M,D)
5.	$x_{i}\leftarrow \arg\max_{x\in X}S\left(x,p(y|x,D)\right)$
6.	$y_{i}\leftarrow f\left(x_{i}\right)$
7.	$D\leftarrow D\cup \{\left(x_{i},y_{i}\right)\}$
8.	End For

**Table 5:** Feature importance ranking.

Order of Importance	Parameter	Importance Score
1	Air temperature	0.74332
2	Wind speed (m·s ^−1^)	0.241356
3	Weight (kg)	0.156544
4	Solar radiance (MJ/m^−2^)	0.057285
5	Air relative humidity (%)	0.046333
6	PM_2.5_ mass concentration (μg·m^−3^)	0.016144
7	PM_10_ mass concentration (μg·m^−3^)	0.013554
8	NO_2_ (ppm)	0.013261
9	CO_2_ (ppm)	0.006566

**Table 6:** MAE values of the model corresponding to different numbers of input features.

Parameter Number	MAE (°C)
1 Air temperature	0.8544
2 Air temperature; wind speed	0.6971
3 Air temperature; wind speed; weight	0.6095
4 Air temperature; wind speed; weight; solar radiance	0.5747
5 Air temperature; wind speed; weight; solar radiance; air relative humidity	0.5326
6 Air temperature; wind speed; weight; solar radiance; PM_2.5_	0.5609
7 Air temperature; wind speed; weight; solar radiance; PM_10_	0.5996
8 Air temperature; wind speed; weight; solar radiance; ...	0.6388
9 Air temperature; wind speed; weight; solar radiance; ...	0.6292

**Table 7:** Hyperparameter optimization results.

Hyperparameters	Value
Convolutional kernel size (n×1)	3 × 1
Number of convolutional layers (*n*)	4
Convolution kernels (*n*)	16
Initial learning rate (*n*)	0.010586
The impact rate of the previous iteration on this iteration (*n*)	0.81753
Regularization factor (*n*)	0.000000037228

**Table 8:** Performance comparison of different methods.

Method	MAE (°C)	MAPE (°C)	RMSE (°C)	$R^{2}$
BP	0.5551	0.0309	0.6831	0.8615
CNN	0.5326	0.0305	0.6599	0.8937
RF–Bayes–BP	0.3895	0.0301	0.4766	0.9206
RF–Random Search–BP	0.3744	0.0208	0.4622	0.9305
RF–Grid Search–BP	0.3569	0.0199	0.4447	0.9365
RF–Grid Search–1D CNN	0.5401	0.0304	0.4865	0.8693
RF–Random Search–1D CNN	0.3672	0.0204	0.4554	0.9384
RF–Bayes–1D CNN	0.3306	0.0176	0.4272	0.9536

**Table 9:** This is a wide table.

Group Number	Sheep Number	Feature	Disease	Range of EBT (°C)	Range of Weight (kg)
$T_{1}$	10	No anomalies	None	15.9–21.9	16.3–41.5
			Diarrhoea		
$T_{2}$	7	Feeble	Rumen flatus	15.3–20.6	21.7–32.7
			Rumen food accumulation		
			Brucellosis in sheep		
$T_{3}$	6	Intermittent	Infectious gastritis	15.8–24.3	17.3–29.3
			Parasitic diseases		
$T_{4}$	17	Persistent	Influenza	15.8–25.5	15.2–47.8

## Data Availability

The datasets presented in this article are not readily available due to institutional confidentiality requirements, as the data are jointly owned by the authors and the Institute of Animal Husbandry, Heilongjiang Academy of Agricultural Sciences. Requests to access the datasets should be directed to zjzshino.1@gmail.com.

## Abbreviations

The following abbreviations are used in this manuscript:

EBT	Ear-Base Temperature
RF	Random Forest
BP	Backpropagation Neural Network
CNN	Convolutional Neural Network
1D CNN	One-Dimensional Convolutional Neural Network
RF–Bayes–1D CNN	Random Forest–Bayesian Optimization–One-Dimensional CNN
TMR	Total Mixed Ration
%DM	Percentage on a Dry Matter Basis
IoT	Internet of Things
GPR	Gaussian Process Regression
AF	Acquisition Function
PDF	Probability Density Function
MAE	Mean Absolute Error
MAPE	Mean Absolute Percentage Error
RMSE	Root-Mean-Square Error
$R^{2}$	Coefficient of Determination
PM_2.5_	Particulate Matter with Aerodynamic Diameter ≤2.5μm
PM_10_	Particulate Matter with Aerodynamic Diameter ≤10μm
CO_2_	Carbon Dioxide
NH_3_	Ammonia
PC	Personal Computer
GPRS	General Packet Radio Service
TCP	Transmission Control Protocol
IP	Internet Protocol
HTTPS	HyperText Transfer Protocol Secure
